# Finding disease outbreak locations from human mobility data

**DOI:** 10.1140/epjds/s13688-021-00306-6

**Published:** 2021-10-19

**Authors:** Frank Schlosser, Dirk Brockmann

**Affiliations:** 1 Department of Physics, Humboldt-University of Berlin, Newtonstr. 15, 12489 Berlin, Germany; 2 Institute for Theoretical Biology, Humboldt-University of Berlin, Philippstr. 13, 10115 Berlin, Germany; 3 Complex Systems Group, Robert Koch-Institute, Nordufer 20, 13353 Berlin, Germany

**Keywords:** Human mobility, Mobile phones, Epidemic spreading, Outbreak detection

## Abstract

Finding the origin location of an infectious disease outbreak quickly is crucial in mitigating its further dissemination. Current methods to identify outbreak locations early on rely on interviewing affected individuals and correlating their movements, which is a manual, time-consuming, and error-prone process. Other methods such as contact tracing, genomic sequencing or theoretical models of epidemic spread offer help, but they are not applicable at the onset of an outbreak as they require highly processed information or established transmission chains. Digital data sources such as mobile phones offer new ways to find outbreak sources in an automated way. Here, we propose a novel method to determine outbreak origins from geolocated movement data of individuals affected by the outbreak. Our algorithm scans movement trajectories for shared locations and identifies the outbreak origin as the most dominant among them. We test the method using various empirical and synthetic datasets, and demonstrate that it is able to single out the true outbreak location with high accuracy, requiring only data of $N=4$ individuals. The method can be applied to scenarios with multiple outbreak locations, and is even able to estimate the number of outbreak sources if unknown, while being robust to noise. Our method is the first to offer a reliable, accurate out-of-the-box approach to identify outbreak locations in the initial phase of an outbreak. It can be easily and quickly applied in a crisis situation, improving on previous manual approaches. The method is not only applicable in the context of disease outbreaks, but can be used to find shared locations in movement data in other contexts as well.

**Supplementary Information:**

The online version contains supplementary material available at 10.1140/epjds/s13688-021-00306-6.

## Introduction

The threat of infectious diseases and epidemics is rising, with new diseases emerging at a seemingly increasing rate [1–3]. The rapid spread of the Sars-Cov-2 virus has recently demonstrated how quickly a communicable, human-to-human transmissible infectious diseases can spread globally [4–6], facilitated by national and international travel patterns. For yet emerging infections, a swift response is crucial to combat a widespread infection, while slow and inefficient measures risk losing control of the event [7].

Many infectious diseases initially spread in a *spatially localized point-source* outbreak. This means that a group of individuals is infected within a short period of time (typically within one incubation period) in a limited spatial area—often associated with a specific point of interest—rather than in many separate, decentralized transmission events spread out over time [8]. For the COVID-19 pandemic, the majority of early cases can be linked to a seafood market in the city of Wuhan, China [9] , which has been described as a localized point-source outbreak [10]. Later clusters often emerged from specific locations where super-spreading events took place, such as dance clubs [11], church services [12] or choir practices [13]. Similarly, past outbreaks of viruses such as Mers, Sars, and Ebola have been linked to super-spreading events or have been centered on specific locations [14–16]. Moreover, there are many other types of diseases where cases occur in a spatially localized point-source outbreak. These include non-communicable infectious diseases, food-based illnesses, environmental hazards (chemical or biological), or even the deliberate release of a biological agent such as anthrax in the context of bioterrorism [17–22].

However, in real scenarios the location of a point-source outbreak is often unknown in the initial phase of the outbreak, and current methods that intend to rapidly identify the outbreak location are tedious in many regards. The predominant method consists of a team of epidemiologists conducting interviews with the infected patients as well as their family, friends or other contacts, trying to manually correlate their movements to find commonalities [23–25]. Such an extensive investigation poses many disadvantages: It is very resource-intensive, requiring a great number of highly-trained staff; It is time-consuming, spanning well over 24 hours, while a swift reaction is paramount at the onset of an epidemic; and finally the highly manual process is error-prone and bears the risk of oversights or false identifications.

There are other established epidemiological approaches to determine the origin of disease outbreaks, including interview-based contact tracing [26–28], transmission chain tracking using virus genomic data [29–31], or tracking of the phylogeographic spread of a virus using genomic sequencing data [32, 33](see [25] for a recent review of methods). Yet, these approaches are in general not applicable immediately after an outbreak occurred, as they rely on the existence of secondary transmissions and established transmission chains, and/or use advanced data sources such as genomic sequencing data, which are not available shortly after an outbreak.

Theoretical models of epidemic spreading on contact networks offer tools for analyzing infectious spreading processes [34–36]. The specific problem of identifying the source of spreading has only been formulated recently [37], leading to a burst of studies on the topic ([38–45], see also [46] for a recent review). However, the proposed methods generally assume a communicable disease and analyze the transmission path to find the source of transmission. They are thus not applicable at the beginning of an outbreak or for non-communicable diseases or hazards. Moreover, many proposed methods require highly processed information, such as the contact network or transmission network, or rely on the computation of complex quantities such as centrality measures or shortest path tress, which are not readily available in a crisis situation [46].

Digital sources of information on human mobility offer a promising new way to automate outbreak location detection [47–50]. Many people carry a mobile phone or similar devices that passively or actively record their movements, offering a reliable account of their recent movement history. Accessing this wealth of data with novel computational methods promises a fast, reliable way of extracting relevant information, such as the origin of an infectious disease outbreak. In the wake of the COVID-19 outbreak, many studies have focused on using smartphones to track transmission chains, and app-based solutions have been implemented in several countries [51–55]. However, these solutions often also rely on the existence of secondary infections and transmission chains by measuring contacts between individuals. In [56], it was instead shown that GPS mobility data can be used to identify the outbreak location in a simulated exercise by correlating the movement of individuals. However, the study used a team of trained specialists manually analyzing the data, and to our knowledge no systematic, computational method has been proposed yet.

Here, we propose a novel method to identify outbreak locations of point-source outbreaks from geo-located GPS movement data of affected individuals as recorded from mobile phones. Our method searches for locations that have been visited by multiple individuals within a short time span and identifies the outbreak locations as the most prominent among them. To the best of our knowledge, this is the first method that only requires unprocessed GPS data to identify the outbreak location. We test the method with regards to its accuracy and robustness to noise, using several datasets of human mobility. The method can easily be extended to the case of multiple outbreak locations, as well as used to estimate the number of outbreak sources if it is unknown. Our method offers a reliable, fast way to locate the origin of an outbreak using otherwise unprocessed data, and can thus be rapidly applied in a crisis situation.

## Inference method

### Scenario definition

The goal of our method is to determine the outbreak origin (both location and time) using the mobility data of affected individuals. We assume that an outbreak has taken place at an outbreak origin $m^{*}=(\boldsymbol{x}^{*},t^{*})$, where $\boldsymbol{x}^{*}$ is the outbreak location and $t^{*}$ the outbreak time. The outbreak has infected a group of *N* individuals, which are all those individuals in the population that were present at the outbreak location at the outbreak time. These are the only individuals affected by the outbreak. Specifically, we assume that no human-to-human transmission and thus no secondary infections have occurred.

The method aims to identify the outbreak location, given only the GPS movement data of the affected individuals in a time frame including the outbreak. For each affected individual $i=1,\ldots ,N$, we are given a movement trajectory $\{ \boldsymbol{x}_{i} (t ) \} $, consisting of pairs of latitude and longitude coordinates measured at discrete time points $t\in \{ t_{0},\ldots ,t_{\mathrm{max}} \} $, where the time frame includes the outbreak time, $t_{0} \leq t^{*} \leq t_{\mathrm{max}}$. In practice, this movement trajectory can be retrieved from mobile phone GPS or similar data sources. The aim of the method is to determine the outbreak origin $m^{*}$ given solely the movement trajectories of the affected individuals.

### Objective function

**Figure 1:**
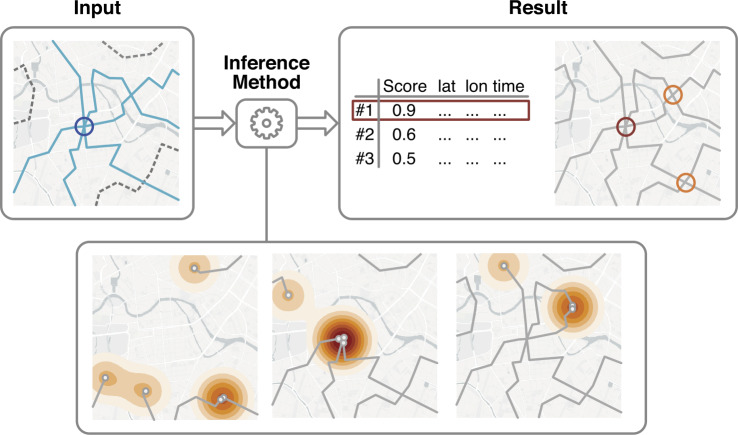
Illustration of the inference method. The input is a sample of GPS trajectories of individuals that have visited the outbreak origin (blue circle). The input sample may also include trajectories of other, unaffected individuals (grey, dashed lines). The method aims to infer location and time of the outbreak from GPS data. To this end, the algorithm identifies occurrences when individuals have been in close proximity of each other by searching for local maxima in the spatial distribution of individuals. The most prominent of these maxima is then identified as the estimated outbreak location

The main idea of our method is to identify the outbreak event as the time when most individuals were in close proximity to each other (see illustration in Fig. 1). We assume that the most prominent common feature of all *N* affected individuals is that they were present at the outbreak location at the same time. Thus, the “closeness” of all individuals reaches a maximum at the outbreak event.

To formalize this notion of closeness, we define the objective function

1 $$ F (\boldsymbol{x},t )=\sum_{i}^{N}f_{i} (\boldsymbol{x},t ), $$

which consists of the sum of individual spatial probability densities

2 $$ f_{i}(\boldsymbol{x},t)=\frac{1}{\sqrt{2\pi \sigma ^{2}}}\exp \biggl(- \frac{ (\boldsymbol{x}-\boldsymbol{x}_{i}(t) )^{2}}{2\sigma ^{2}} \biggr). $$

The spatial probability density $f_{i}(\boldsymbol{x},t)$ is a normal distribution centered around the position $\boldsymbol{x}_{i}(t)$ (a vector of longitude/latitude coordinates) of individual *i* at time *t*. If individuals are in close proximity to each other, their spatial probability densities overlap and the value of the objective function increases. The objective function $F (\boldsymbol{x},t )$ thus measures the closeness of all individuals, or how shared a location is among them, and reaches its theoretical maximum if all individuals are at exactly the same location $\boldsymbol{x}_{i}(t)=\boldsymbol{x}_{c}$. In practice, we expect it to reach a maximum at the true outbreak location and time.

To make the scaling of the objective function more intuitive, we instead use the *score*

3 $$ S (\boldsymbol{x},t )=\frac{1}{Z}F (\boldsymbol{x},t ), $$

which lies in the range $S\in [0,1]$ using the normalization constant $Z=\sqrt{2\pi \sigma ^{2}}/N$. The maximum of $S=1$ implies that all *N* individuals were present at exactly the same coordinates at a given time, while a vanishing score would imply that all individuals were far apart from location ***x***. A useful property of this definition is that we can read the estimated number of individuals present at a location from the score, $N_{\mathrm{est}}(\boldsymbol{x},t)=NS (\boldsymbol{x},t )$.

The standard deviation *σ* is the only free parameter in Eq. (3), which signifies the strength of the spatial error in the location measurement, or, alternatively, the leeway we give in the desired overlap of the spatial kernels. We find that this parameter has little influence on the inference accuracy and that a wide range of values work well. Here, we use a value of $\sigma =1.57\mathrm{e}{-5}$ in radians, which corresponds to approximately $100~\mathrm{m}$ (see Additional file 1 Sect. 2 for more details).

### Inference of the outbreak origin

Finally, the outbreak origin can be inferred by finding maxima of the score function $S (\boldsymbol{x},t )$ in both space and time. As described above, the score *S* has local maxima if individuals were close to each other spatially at one point in time, and we expect *S* to have a global maximum at the true outbreak location $\boldsymbol{x}^{*}$, which all individuals visited at the time $t^{*}$. Thus, the estimate for the outbreak origin, $\widehat{m}=(\widehat{\boldsymbol{x}},\widehat{t})$, is given by the global maximum of $S(\boldsymbol{x},t)$ over space and time,

4 $$ \widehat{m}= (\widehat{\boldsymbol{x}},\widehat{t} )=\arg \Bigl( \max _{t} \Bigl(\max_{\boldsymbol{x}}S (\boldsymbol{x},t ) \Bigr) \Bigr). $$

We determine the global maximum of $S(\boldsymbol{x},t)$ using a numerical optimization algorithm: For each time point *t*, we construct the function $S(\boldsymbol{x},t)$ from the given locations $\boldsymbol{x}_{i}(t)$ of individuals, calculate the maximum of $S(\boldsymbol{x},t)$ numerically using a grid search, and finally determine the global maximum *Ŝ* and the corresponding estimated outbreak origin $\widehat{m}= (\widehat{\boldsymbol{x}},\widehat{t} )$ as the maximum over all time points *t* (see Additional file 1 Sect. 1 for a definition of the algorithm).

The assumption that the outbreak origin is the unique global maximum of *S* is only true if there is no other location that was visited by all individuals simultaneously—which might happen by chance, or due to errors in the data. Such other gatherings would then “mask” the true outbreak location and are the main limiting factor of the accuracy of our approach. We investigate their influence in detail in Sect. 4.

### Extension to multiple outbreaks

The inference method can easily be expanded to the case of multiple outbreak origins. In this extended scenario, we assume that multiple outbreak events $m^{*}_{1}, m^{*}_{2}, \ldots, m^{*}_{M}$ took place, which are spatially and temporarily independent of each other. Each of the *N* affected individuals in the sample were present at either one (or multiple) of the outbreak events.

In this case, we expect that there are multiple distinct maxima in the score $S(\boldsymbol{x},t)$ corresponding to the different outbreak origins, as at those times a considerable subgroup of the *N* individuals were in close proximity of each other. To detect these origins, we change the algorithm to not only save the global maximum of $S(\boldsymbol{x},t)$, but all local maxima, and rank them by their score *S*, which yields a list of location sorted by their likelihood to be an outbreak origin (see illustration 1). If the number of outbreak events *M* is known, these are estimated as the top location *M* locations sorted by score. If the number of outbreak events is unknown, the scores can be used to estimate it, see Sect. 4.4.

Creating this list is useful in case of only one outbreak origin, as well, because it allows experts to easily check the results of the method manually. In cases where the estimate of the outbreak origin is wrong, the true outbreak origin is still very likely to be among the top scoring locations. Lastly, note that when saving all local maxima, the resulting list often contains the same location (or locations very close to it) multiple times. To account for this, we cluster and aggregate maxima that are spatially close (see Additional file 1 Sect. 1 for details).

### Extension to include non-simultaneous visits

In many scenarios, an outbreak does not only occur at one point in time, but can be stretched over a prolonged timespan and may cause infections at different points in time. An example would be diseases that are transmitted by shared surface contact and smear infections. Infected individuals then do not have to have been present at the outbreak location at the same time, but might have visited it at different times. In our framework, we are then interested in finding movement trajectories that visited a certain location sometime in their movement history.

We can easily extend our method to include such non-simultaneous visits by adjusting the objective function in Eq. (1) to consider locations at multiple points in time, resulting in the “time-smeared” objective function

5 $$ F' (\boldsymbol{x},t )= \sum _{t'=0}^{T_{\mathrm{max}}} \sum_{i}^{N}f_{i} (\boldsymbol{x},t )\cdot g\bigl(t, t'\bigr), $$

with the temporal kernel

6 $$ g\bigl(t, t'\bigr) = \mathrm{exp} \biggl( -\frac{(t-t')^{2}}{2\sigma _{t}^{2}} \biggr), $$

which means that at each point in time *t*, not only are the locations of all individuals at this time considered in the search for possible outbreak origins, but also their positions at adjacent times. The temporal variance $\sigma _{t}^{2}$ determines how many time points are considered, and should be set depending on how long one expects the outbreak to have lasted.

## Datasets

### Data format

The inference method uses a set of individual mobility trajectories $\{ \boldsymbol{x}_{i} (t ) \} $ as input, in the format of time-stamped location measurements. The data can stem from a variety of sources as long as it follows this basic format.

Although there are no specific requirements on the data source, there are some soft requirements regarding the data resolution to ensure an adequate performance of the method. The spatial resolution of the data should be fine-grained enough to distinguish separate locations. GPS data derived from smartphone devices is best suited as it offers high accuracy [48]. Data derived from cell tower logs can also be used, especially in urban environments where cell tower locations are close, although the spatial accuracy is lower in general. Regarding the temporal resolution, it is clear that a finer resolution improves the performance of the method. In this study, we use a resolution of 15 minutes for all datasets. As a minimum, the temporal resolution should be high enough to record all subsequent stationary locations of an individual.

### Empirical datasets

We test the method using a variety of empirical and synthetic datasets (detailed descriptions of the dataset can be found in the Additional file 1 Sect. 3). We use two empirical datasets that were obtained from GPS devices: The datasets CNS and GEOLIFE.

The first empirical dataset CNS was gathered as part of the Copenhagen network study [57]. It includes the GPS movement data of 689 students in Copenhagen, recorded using smartphones and cell tower location data, at an interval of 15 minutes.

The second empirical dataset GEOLIFE was collected by Microsoft Research Asia in the Geolife project by [58–60]. After pre-processing (see Additional file 1 3.1 for details), the resulting dataset contains trajectories of $N=75$ individuals. Due to the relatively small size of this dataset, we limit the outbreak size to a maximum of $N=5$ individuals in our measurements. We found that a larger outbreak size leads to fewer and fewer valid outbreak scenarios (where *N* people were present in the same location), introducing strong systematic biases (see Additional file 1 for a more detailed explanation).

### Synthetic datasets

In addition to the empirical datasets, we generate movement trajectories using three well-known human mobility models, covering different modeling approaches. We use algorithms published in previous studies and take care to use the default parameters whenever possible (see Additional file 1 Sect. 4). We thus generate the datasets dEPR, sOD and dOD.

First, we create the dEPR dataset by implementing a gravity-law like mobility model, namely a variation of the exploration and preferential return (EPR) model [61]. In the EPR model, individuals explore new locations or return to previous locations. At each time step, individuals will either explore a new location or return to a previous location. When choosing which location to return to, locations with a high visitation frequency are chosen preferentially. This mechanism results in a realistic individual location frequency distribution when compared to real data. We use an extension of the original model known as the density-EPR (d-EPR) model described in [62], where individuals choose new locations from a given set of locations, depending on the distance to the current location (following a gravity-like law) and the weight of the new location. Accounting for this location density has been shown to result in a more heterogeneous, realistic spatial distribution. The set of locations we use for the d-EPR model is extracted from geolocated Twitter data from the Berlin area (see Additional file 1 Sect. 5.1).

Second, we implemented two variants of an agent based simulation of mobility based on origin-destination (OD) matrices to generate the datasets sOD and dOD. At its core, the model uses an OD matrix containing the recorded statistical flows of individuals between spatial cells to simulate individual movements between spatial cells. We use a set of OD mobility flows aggregated by a mobile phone provider from cell tower logs in the area of Berlin, Germany (see Additional file 1 Sect. 5.2). When an individual travels, it chooses its target spatial cell proportional to the flows from its current cell at the given time.

To determine the location of individuals within those spatial cells, we use two different approaches. In one variant, we use the common approach of choosing the location randomly in the space of each cell, leading to the sOD (spatial-OD) dataset. In the other variant, we choose the location within each cell from the location density extracted from the Twitter data, similar to the d-EPR model, thus creating the dOD (density-OD) dataset.

For each synthetic dataset, we simulate the movements of 10,000 individuals over the course of one month. In total, we use the three synthetic datasets dEPR, sOD and dOD together with the empirical datasets CNS and GEOLIFE.

## Results

### Generation of outbreak scenarios

To test the accuracy of our method, we first simulate an outbreak scenario with an outbreak origin $m^{*}$ and then apply the inference method to it. The task of the inference method is to estimate the outbreak origin *m̂*. We assess the methods’ accuracy by comparing the inference result *m̂* to the true outbreak origin $m^{*}$.

To generate an outbreak scenario for a given movement dataset, we first choose a random outbreak origin $m^{*}=(\boldsymbol{x}^{*},t^{*})$ among all locations and times present in the dataset. Then, we choose a sample of *N* individuals from the dataset which have been within $50~\mathrm{m}$ of the outbreak location $\boldsymbol{x}^{*}$ within $30~\mathrm{min} $ around the outbreak event time $t^{*}$. If none or less than *N* individuals have been to the outbreak origin, we choose a new random outbreak origin.

The input for the inference method is then the set of movement trajectories $\{ \boldsymbol{x}_{i}(t) \} _{i=1...N}$ of the *N* sampled individuals. We limit the trajectories to a time span of 7 days, centered around the outbreak event time. This a realistic time span in a practical scenario, where one can assume that the timespan where the outbreak might have occurred can be narrowed down to 7 days. We found the length of the time span to have limited effect on the accuracy of the method, although in general the accuracy decreases with increasing time span.

### Inference accuracy depending on sample size

The first question we examine is: How much data is necessary to identify the outbreak origin $m^{*}$ among all possible locations with sufficient accuracy? We expect that with larger sample size *N*, the outbreak origin $m^{*}$ is easier to identify as it is always visited by all *N* individuals, while other locations are visited only when individuals meet by chance. In the words of our methodology, we expect the global maximum of the score function *S* to be more distinct with increasing *N*. To test this hypothesis, we choose a single outbreak origin $m^{*}=(\boldsymbol{x}^{*},t^{*})$, select a sample of *N* trajectories, estimate the outbreak origin $\widehat{m}=(\widehat{\boldsymbol{x}},\widehat{t})$ as the location with the highest score *S*, and compare it to the actual outbreak origin $m^{*}$.

To quantify the methods’ accuracy, we calculate the *distance error* $\Delta x= \Vert \widehat{\boldsymbol{x}}-\boldsymbol{x}^{*} \Vert $ between the true and estimated location. We deem the inference correct if the distance error is smaller than $100~\mathrm{m}$, so that the *location accuracy* (i.e. the probability of correct location inference) is $P_{\mathrm{loc}}=P(\Delta x<100~\mathrm{m})$. Similarly, we define the *temporal error* Δ*t* as the difference between the true and estimated outbreak times, $\Delta t= \vert \widehat{t}-t^{*} \vert $, and deem the inference correct if the temporal error is smaller than 1 hour, so that the *time accuracy* is $P_{\mathrm{time}}=P(\Delta t<1\mathrm{h})$.

**Figure 2:**
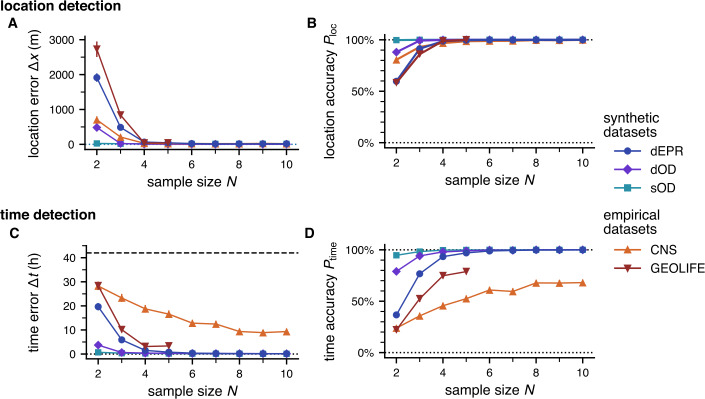
Accuracy of the outbreak detection, depending on the number of individuals *N* in the sample. In these and further plots, results are averaged over 1000 measurements for each dataset and sample size *N*. Error bars indicate the standard error. **A** The distance error Δ*x* between true and estimated location decreases quickly with growing sample size *N*. **B** The method is able to infer the correct outbreak location with high accuracy $P_{\mathrm{loc}}$, even for a small number of input trajectories. **C** The temporal error Δ*t* decreases fast for the synthetic datasets, but less so for the empirical datasets, which we attribute to repeated behavioral patterns in the datasets’ populations (see main text). The dashed line at $\Delta t=42\mathrm{h}$ marks the expected error for a random guess of the outbreak time within the sample interval of 7 days. **D** The accuracy $P_{\mathrm{time}}$ of the time inference increases more slowly, and the accuracy is generally lower for the empirical datasets, which we again attribute to repeated behavioral patterns

We find that the method is very accurate in finding the true outbreak location $\boldsymbol{x}^{*}$, even for small sample sizes (see Fig. 2). The distance error Δ*x* decreases rapidly with growing sample size *N*, as expected. For $N=4$, the method already identifies the outbreak location with close to $P_{\mathrm{loc}}\approx 100\%$ accuracy on all datasets.

Similar to the outbreak location, the method is able to infer the outbreak *time* $t^{*}$ with good accuracy (see Fig. 2), although a higher sample size *N* is required in general. In particular, we observe a distinct difference between the synthetic and empirical datasets: While the temporal error Δ*t* decreases fast for the synthetic datasets, it decreases noticeably slower on the empirical datasets CNS and GEOLIFE. Similarly, the accuracy of the time inference $P_{\mathrm{time}}$ saturates at lower levels for the empirical datasets.

Overall, we find that inferring the outbreak time is more difficult as individuals tend to revisit locations in their trajectory (which are part of their routine, or their set of commonly visited locations) multiple times. These repeated gatherings can mask the true outbreak event, so that more data is required to pinpoint the “correct” gathering of individuals. The effect of repeated visits is stronger in the empirical datasets, as they represent subpopulations (students and lecturers) with many shared locations and shared, repeating time schedules; see a more detailed discussion in Sect. 5.

### Accuracy for multiple outbreak locations

The inference method can easily be expanded to the case of multiple outbreak origins $m_{1}^{*},m_{2}^{*},\ldots,m_{M}^{*}$ by not only looking at the location with the highest score *S*, but at a list of top-scoring locations, as detailed in the method Sect. 2.

To test the method for multiple locations, we adapt our scenario generation setup: We choose the first outbreak origin $m_{1}^{*}$ as usual, and all further outbreak origins $m_{i}^{*}$ with $i=2,\ldots,M$ in the same way with the added condition that their outbreak time has to lie within the 7 day time window around the first outbreak, in order to be part of the input sample. The input sample then consists of a total of $N=\sum N_{i}$ trajectories, with $N_{i}$ trajectories chosen from each of the outbreak origins $m_{i}$.

To judge the accuracy of the method when inferring multiple outbreak origins, we look at the $M'$ estimated origins with the highest score *S*, $\widehat{m}_{j}$ with $j=1,\ldots,M'$. We deem the inference correct if all true outbreak origins are included in the set of estimated locations, $\{m_{i}^{*}\}\subset \{ \widehat{m_{j}} \} $. Note that we relax the criterion for accuracy for multiple locations by defining $M':=2M$, i.e. the *M* true outbreak locations have to be among the top 2*M* estimated locations. Otherwise, if we would choose $M'=M$, the true outbreak origins would have to correspond exactly to the top *M* estimated locations, which would be increasingly unlikely for higher *M* and thus introduce an error that grows with *M*, stemming only from this unrealistically strict requirement. The relaxed criterion means that we allow for *M* false positives among the output, which we in practice expect to be identifiable in a manual inspection of the 2*M* locations proposed by the algorithm.

**Figure 3:**
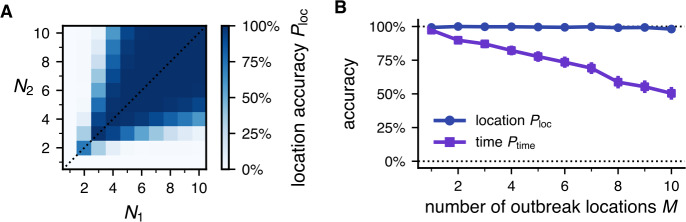
Accuracy of the inference method for multiple outbreak origins. **A** For multiple origins (here $M=2$), the location accuracy $P_{\mathrm{loc}}$ increases with sample sizes $N_{1}$ and $N_{2}$, especially if a similar number of samples is available for all origins ($N_{1} \approx N_{2}$, dotted line). However, if more trajectories stem from one origin instead of the other ($N_{1} \neq N_{2}$, off-diagonal entries), the accuracy actually decreases as the overrepresented location masks the other locations. **B** The accuracy of the location prediction is approximately independent of the number of outbreak locations *M*, while the time accuracy decreases. Sample size is $N_{i}=4$ for all origins *i*. Results are shown for the dEPR dataset

We test the inference method for multiple outbreak origins with varying sample sizes $N_{i}$. In Fig. 3, we show the results for $M=2$ outbreak origins, but the qualitative features stay the same if extended to more outbreak origins. We find that the method is able to detect multiple outbreak origins with high accuracy, similar to the result for a single outbreak origin. In general, the accuracy increases with bigger sample sizes $N_{i}$, analogous to the result for one location. For $N_{1}=N_{2}=4$, the inference is able to find both origins in 99% of cases. Results are shown for the dEPR dataset, but we find no qualitative differences to other datasets.

Interestingly, if we fix the sample size for one location and increase the sample size of the other location, the accuracy actually decreases, despite more data being available for the inference. For such unbalanced datasets, where most trajectories belong to one of the locations, the dominant location masks the other gathering sites, making them more difficult to find for the algorithm.

### Estimating the number of outbreak locations

In a practical scenario, it is likely that the number of outbreak origins *M* is unknown initially—if, for instance, the outbreak events are undetected, but at a later time affected individuals show up in medical care facilities. In this case, it is important to first find out *how many* outbreaks took place, in addition to their location and time.

We find that our method can reliably estimate the number of outbreak origins *M̂* from the distribution of scores *S* by utilizing characteristic features of the distribution. Here, we assume that the outbreaks $m_{i}$ are of the same size, i.e. affecting the same number of individuals $N_{i}=c$, adding up to the total sample size $N=c*M$.

**Figure 4:**
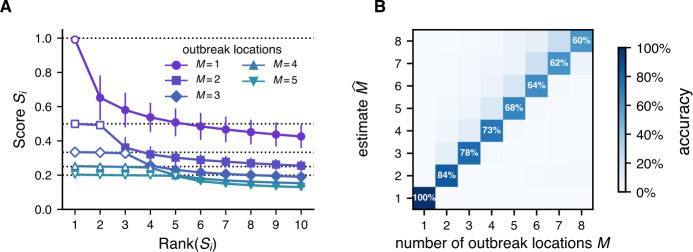
Estimation of the number of outbreak origins from the score distribution. **A** The scores $S_{i}$ of the 10 highest-scoring locations, for a varying number of outbreak origins *M*. We find that the top *M* locations corresponding to the true outbreak origins have a distinctly high score (hollow symbols), at around the expected values of $E(S_{i})=1/M$ (dotted lines). The scores of further locations follow an exponentially decaying distribution. Error bars indicate the standard deviation. **B** Based on the characteristic form of the score distribution, we define an estimation heuristic for the number of outbreak locations, $P(\widehat{M}=M)$ (see main text). The heuristic correctly identifies the true number of outbreak sources *M*, where the accuracy decreases with rising number of outbreak origins and tends to overestimate the number of outbreak origins

Following from the definition of the score *S*, the expected score $E(S_{i})$ of an outbreak origin $m_{i}$ is the fraction of individuals in the sample that visited that outbreak origin, $E(S_{i})=N_{i}/N$. If the outbreak sites were visited by an equal amount of individuals, all origins thus have the expected score $E(S_{i})=1/M$. In our scenarios, we indeed find that the *M* highest-scoring locations, which correspond to the actual outbreak origins, have a very similar score of around the expected value $1/M$ (see Fig. 4A). The remaining locations with $i>M$ follow an exponentially decaying distribution.

We can exploit the characteristic and predictable structure of the score distribution to estimate the number of outbreak origins *M* in a sample using a simple heuristic. As we see in Fig. 4A, the empirical distribution of scores $S_{i}$ has a discontinuity at the true number of outbreak locations $i=M$, where the second derivative of the distribution is negative while otherwise being positive. Using this observation, we estimate the number of outbreak origins *M̂* as

$$ \widehat{M}=\arg _{i} \Bigl(\min_{i}\Delta ^{2}S_{i} \Bigr), $$

with the second order difference quotient

$$ \Delta ^{2}S_{i}=S_{i-1}-2S_{i}+S_{i+1}. $$

The heuristic fails for the special edge case of $M=1$, where the second order derivative is non-negative. However, we find that we can reliably test for this case by setting $\widehat{M}:=1$ iff $S_{1}>0.8$, i.e. when one location clearly dominates the score distribution.

We find that the heuristic well predicts the number of actual outbreak origins, see Fig. 4B, where the accuracy decreases with rising number of outbreak origins *M*.

### Robustness to noise and secondary infections

**Figure 5:**
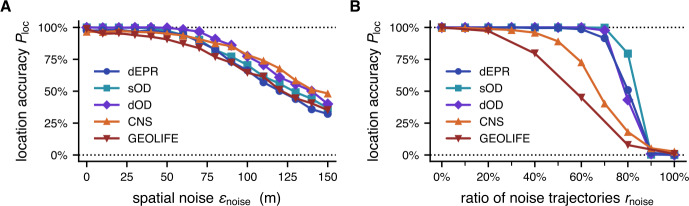
Robustness of the method to noise in the input data and secondary infections. **A** We apply Gaussian noise with standard deviation $\epsilon _{\mathrm{noise}}$ to the location data of $N=4$ individuals. The accuracy decreases only after unrealistically high levels of noise are applied, compared to the mean smartphone GPS accuracy of $4.9~\mathrm{m}$ [63]. **B** We test the robustness to individuals in the sample that were not present at the outbreak origin, for example due to secondary infections. Starting with a sample of $N=10$ individuals, we replace a fraction of $r_{\mathrm{noise}}$ trajectories with random individuals from the dataset. The inference method is robust to high amounts of noise or secondary infections for the synthetic data, and moderate amounts for the empirical datasets

Finally, we tested the robustness of the inference method with regards to various sources of noise in the datasets, as well as to the influence of secondary infections. The simplest and most likely form of potential noise in our scenario is a statistical error in either the location or time measurement of the movement trajectories $\boldsymbol{x}_{i}(t)$. In practice, we don’t expect a significant error in the *time* measurement, as data collection devices such as mobile phones are able to measure time with high precision (compared to the much longer time scale of the outbreak event itself), and we will therefore neglect this error source here. In contrast, the *location* measurement is likely to be influenced by noise, considering for instance that the average error of a smartphone GPS signal is 4.9 meters [63]. Regarding the *location* measurement, we tested the method by applying a Gaussian noise with a varying standard deviation $\epsilon _{\mathrm{noise}}$ to the locations in the input data for $N=4$ individuals (see Fig. 5A). We find that the accuracy of the method only noticeably starts to decrease at noises greater than about $\epsilon _{\mathrm{noise}}>50~\mathrm{m}$, which is a magnitude greater than the error that is realistically to be expected. For a greater sample size than $N=4$, we expect the error to be even smaller.

We also test the robustness of the method to secondary infections and falsely labelled input data. When given a sample of trajectories, so far we implicitly assumed that all individuals actually visited the outbreak origin, which might not be the case in practice. Over time, the number of secondary infections in the population will increase, and we can expect that increasingly fewer individuals in the sample have actually been present at the original outbreak. In addition, misdiagnosis of a patients symptoms or other processing errors can lead to individuals being erroneously included in the sample. To test the robustness of our method to these sources of error, we run simulations for one outbreak origin with $N=10$ trajectories where we include a varying fraction $r_{\mathrm{noise}}$ of *noise trajectories* in the input data, i.e. trajectories of random individuals from the dataset that have not necessarily been present at the outbreak origin (such noise trajectories are displayed in Fig. 1).

We find the method to be robust to moderate amounts of noise trajectories such as introduced by secondary infections, see Fig. 5B. For the CNS and GEOLIFE datasets, the inference method is able to determine the correct outbreak location with 50% probability for a fraction of $r_{\mathrm{noise}}\approx 65\%$ noise trajectories. For the simulation data, we observe a more distinct threshold for a higher amount of noise at $r_{\mathrm{noise}}\approx 80\%$. The high robustness to noise input demonstrates that the inference method is able to pick up the outbreak signal reliably, especially for the synthetic data, where the inference only breaks down when the true signal itself becomes too weak. Again, we attribute the different behavior of the synthetic and empirical datasets due biases in the populations depicted in the CNS and GEOLIFE datasets that hinder the inference, see discussion.

## Discussion

In this paper we have introduced a novel method to identify outbreak locations of point-source outbreaks of infectious diseases, using GPS mobility data of affected individuals. We have shown that the method is able to identify the outbreak location reliably, requiring only as little data as trajectories of $N=4$ individuals. The rapid applicability is a considerable improvement on the currently used method of manual data analysis, for which it has been found to take up to 6 hours to identify the outbreak location if conducted by a team of epidemiologists focused on the task [56]. Even if the location identified by the method is not the correct outbreak location, it is very likely that the true outbreak location is among the top-scoring locations, such that a manual inspection can quickly confirm the results of the algorithm. We have also shown that the method is robust to noise in the input data. Noise in the GPS signal does not have a significant effect on the accuracy, at least at levels that can be expected in real data. Falsely labelled input data, for example due to inclusion of not-affected individuals in the data sample, can decrease the accuracy of the method at moderate to high levels of noise.

In multiple instances we find interesting differences in the algorithms’ performance between the synthetic and empirical datasets, which we attribute to specific characteristics of the empirical datasets. In the empirical datasets, it is harder to determine the correct outbreak time, and including falsely labelled input data lowers the accuracy sooner than for the synthetic datasets. We attribute these observations to the makeup of the populations in the CNS and GEOLIFE datasets: Both datasets contain the data of narrow subpopulations of students and faculty at the same universities (a detailed description of the datasets and their spatial distributions can be found in the Additional file 1 Sect. 3). The shared schedule and repeated visits to common locations in these datasets make it more difficult to determine the precise outbreak time. Likewise, choosing a random person from the dataset as a “false” input trajectory is likely to choose an individual whose movement history overlaps with other individuals in the sample, more so than that of an individual from the general population would. Thus, we expect that the synthetic datasets better represent the algorithms’ performance in a general population, but note that outbreaks occurring in narrow subpopulations can hinder the inference.

Our approach requires high-resolution GPS data of individuals affected in the outbreak. This type of data is by its nature highly sensitive and difficult to obtain. Different approaches to gather the required data might include: Asking affected individuals to “donate” their data, using emergency protocols to legally request the data in the context of the epidemiological response, and/or setting up necessary agreements with data providers beforehand. The approach also hinges on the fact that enough affected individuals have a device that records their movements, which might not be the case. This limitation could be circumvented by using movement data collected by the telecommunication providers itself, such as cell tower logs, which only requires the individuals to possess a mobile phone, but not to actively record their data. We expect that the method can be applied to data extracted from cell tower logs without modification, although the spatial accuracy can be expected to decrease.

On the methodological side, we point out that our method neglects secondary infections and is thus mostly applicable in the early stages of an infection event, although we show that the method is still reliably for a moderate amount of secondary infections in the population, see Sect. 4.5. However, as time passes and more secondary infections occur, other epidemiological methods become available as discussed in the introduction.

The inference method could potentially be extended by including secondary and further infections and by taking into account contacts between individuals and models of epidemic transmission. Further, we applied our method only to outbreaks occurring at one point in time here, but as shown in Sect. 2.5 the method can easily be extended to outbreaks occurring over a span of time. Lastly, we think that there are promising ways in which our method could enhance other methods of outbreak detection. For instance, bluetooth-based contact tracing—which has found widespread usage during the COVID-19 pandemic [64]—could potentially be improved by incorporating spatial GPS information as processed by our method, for example by correlating bluetooth-contacts with spatial proximity as measured here.

Our method is the first to offer an out-of-the-box, simple approach to identify outbreak locations in realistic scenarios. It can be easily and quickly applied in a crisis situation, improving greatly on previous manual approaches. Moreover, the method does not rely on any disease dynamics. It is thus not only applicable in the context of infectious diseases, but can be used to find shared locations in movement data in other contexts as well. We hope that future work further improves on the capabilities of the proposed method, and that more novel methods are developed with harness the potential of digital data sources for epidemic control.

## Supplementary Information

Below is the link to the electronic supplementary material.

The synthetic mobility datasets generated and analysed in this study are available in the OpenScienceFramework (OSF) repository https://osf.io/3rzh8/. The implementation of the outbreak detection method (in Python) which used in this manuscript is available at https://github.com/franksh/outbreak-detection. The empirical mobility datasets are available from the original authors, see Additional file 1 Sect. 6 for details. (PDF 2.0 MB)
