# Supplementary material for: Finding disease outbreak locations from human mobility data
Source: EPJ Data Sci. 2021 Oct 19;10(1):52. doi: 10.1140/epjds/s13688-021-00306-6 (PMC8525067; doi:10.1140/epjds/s13688-021-00306-6)
Supplement: Supplementary file 1 — The synthetic mobility datasets generated and analysed in this study are available in the OpenScienceFramework (OSF) repository https://osf.io/3rzh8/. The implementation of the outbreak detection method (in Python) which used in this manuscript is available at https://github.com/franksh/outbreak-detection. The empirical mobility datasets are available from the original authors, see Additional file 1 Sect. 6 for details. (PDF 2.0 MB) [file 13688_2021_306_MOESM1_ESM.pdf]

SUPPLEMENTARY INFORMATION

Finding Disease Outbreak Locations from Human Mobility Data

Frank Schlosser[1,2*] and Dirk Brockmann[1,2]

*Correspondence: frank.schlosser@hu berlin.de
[1]Robert Koch Institute, Nordufer 20, 10115, Berlin, Germany
Full list of author information is available at the end of the article

1 Outbreak detection method

Here we provide a detailed description of the method used to detect outbreak locations from human mobility data. The method is given as a pseudo-algorithm (see algorithm 1) and is available as a implementation in Python at https://github.com/franksh/outbreak-detection, which was used to conduct the measurements in the manuscript.

As input, the method uses a set $\mathcal{X} = \{X_i\}$ of mobility trajectories X_i of $i = 1, ..., N$ individuals. Each mobility trajectory is X_i a set of observations of locations x_j recorded at times $t_j = t_0, ..., T_{\max}$ at intervals of $\Delta t = t_{j+1} - t_j$. In practice, the method can also be applied if the intervals Δt are irregular, or if the recording times t_j vary between individuals, as long as the core inference (i.e. determining the maximum of the score function) is run for all times when the position of any individual in the dataset changes. For ease of notation, we assume regular intervals Δt and the same recording times t_j for all individuals here. Besides the mobility trajectories $\mathcal{X}$, the method uses the spatial variance σ as an input parameter (see section 2 for more details on the choice of σ).

The core loop of the outbreak detection method is the following (see algorithm 1): For each time $t = t_0, ..., T_{\max}$, we determine the spatial maximum x^* of the score function $S(x, \sigma, X')$, where X' is the set of the locations of all individuals at time t. This location $\ell = (x^*, t, S^*)$ is then added to the list of candidate locations L. Determining the spatial maximum of $S(x, \sigma, X')$ can be done by performing a grid search within the spatial bounds of the dataset. In practice, we find that the global spatial maximum can reliably be found by starting a gradient descent at each of

the locations X' of the individuals at time t, although we provide no proof that this guarantees finding the global maximum.

The output of the algorithm is a list of outbreak location candidates L, which can be sorted by their score S_ℓ. The proposed outbreak location is then the location L^* with the highest score S_ℓ^*. A ranked list of the highest-scoring locations can also be reviewed manually, with the actual outbreak likely among them.

Before determining the estimated outbreak origin L^* we perform a final intermediary data-processing step, namely a spatial clustering of all locations in the set L. This clustering is useful as, in general, many locations are found multiple times, for example at adjacent time points. We use the `DBSCAN` algorithm with the parameter $\sigma = 100$m as the maximum distance between two observations for them to be considered part of the same location.

Algorithm 1: Outbreak detection algorithm

Data: The set $\mathcal{X} = \{X_i\}$ of mobility trajectories X_i of $i = 1, ..., N$ individuals, where each mobility trajectory $X_i = \{(x_j, t_j)\}$ is a set of observations of locations x_j recorded at the times $t_j = t_0, ..., T_{\max}$ at intervals $\Delta t = t_{j+1} - t_j$.

Input: The mobility trajectories $\mathcal{X}$ and the spatial variance σ.

Output: A set of likely outbreak locations $L = \{(x_j, t_j, S_j)\}$ including the spatial location x_j, time t_j, and score S_j of the location.

Procedure FIND–OUTBREAK–LOCATIONS($\mathcal{X}, \sigma$):

```
    L ← {};                                        // Outbreak locations
    t ← t_0;
    while t < T_max do
        // Get current locations of individuals
        X' ← {x_j ∈ 𝒳 | t_j = t};
        // Determine location with maximum score
        x* = max_x(S(x, σ, X'));
        S* = S(x*, σ, X');
        // Save location
        L ← L + (x*, t, S*);
        // Update time
        t ← t + Δt;
    end
    // Cluster locations
    L ← DBSCAN(L, σ);
    return L;
// Score function
def S(x, σ, {x_i}):
    return N/√(2πσ²) Σ_i^N 𝒩(x; x_i, σ);           // Sum of normal distributions
```

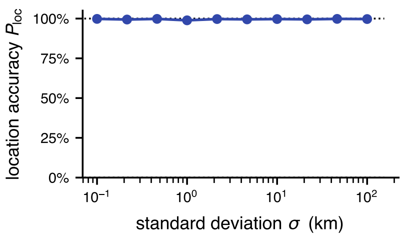

Figure 1 Influence of the standard deviation σ on the detection accuracy. We use the scenario of a single outbreak origin with a sample size of $N = 5$ individuals, for which we expect close to 100% accuracy (see results in main manuscript). We find little dependence of the accuracy on the value of σ. Results are shown for the `dEPR` dataset for $1,000$ measurements at each value.

2 Influence of σ on the detection accuracy

The standard deviation σ is the only free parameter in the score function $S(x, \sigma, \{x_i\})$. It is the standard deviation of the spatial probability kernel that is placed at each individuals' position (see also eq. 2 in the main text).

We find that the value of the σ has little impact on the accuracy of the outbreak detection method, see Figure 1. We test the method for a range of values of σ, ranging from 100m to 100km, which we translate from cartesian to radial coordinates using the formula

$$\sigma = \sigma_{\text{radians}} = \sigma_{\text{cartesian}} \times r_{\text{earth}}$$

with the earth radius $r_{\text{earth}} = 6371.0$ km.

The limited influence of σ on the method is not surprising as it does not influence the location of the maxima in the score function S (or the objective function F), but mainly influences the efficiency of the numerical optimization.

3 Empirical datasets

3.1 CNS dataset

The `CNS` dataset is a subset of the data collected during the Copenhagen Network Study in 2014. In the study, a group of 1000 students were provided smartphones and social sensors. The data they generated was collected, including calls and text messages, social media usage and face-to-face contacts. The location was measured using the GPS measurement of the smartphone sensor or the cell tower location, as available. The accuracy collected position can vary between a few meters for GPS

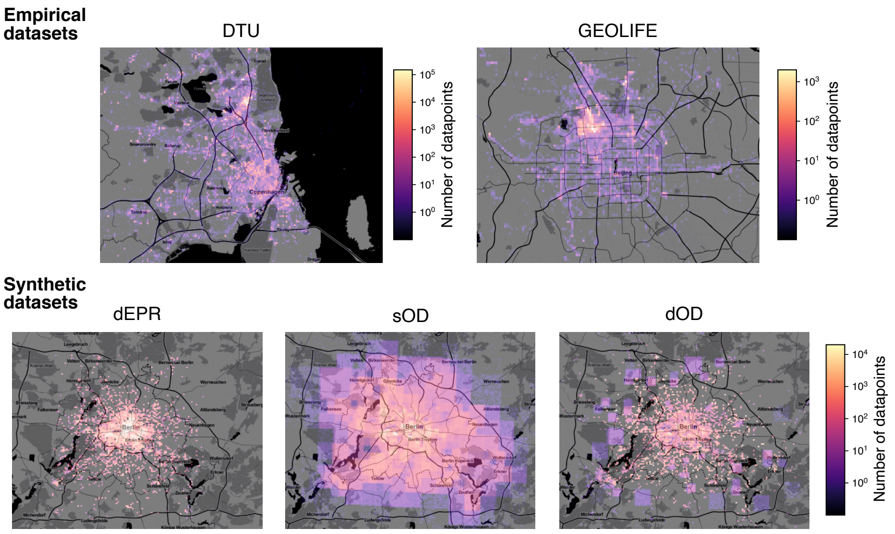

Figure 2 Heatmaps of the mobility datasets. Colors indicate the number of datapoints in each spatial bin, where each datapoint is one time-stamped geo-location of a user or simulated individuals. The spatial distributions of the empirical datasets highlight the specific subpopulations depicted by those datasets: The heatmaps exhibit maxima at the locations of the Technical Unversity of Copenhagen and the Beijing Technical University for the `DTU` and `GEOLIFE` datasets, respectively (upper center and upper left regions of the map). For the synthetic datasets, the distributions highlight methodological differences. In the `dEPR`-model, individuals are placed at locations extracted from Twitter data, while in the `sOD`-model the location is randomized in the spatial cells of the origin-destination matrix. The latter distribution displays the structure of the cells, and the coordinates encompass a wider area. The `dOD` dataset shows combined aspects of both former distributions, as it generally uses Twitter data but falls back on randomized coordinates if no Twitter locations are available within a cell.

locations, to hundreds of meters for cell tower location, where 90% of the samples have a reported accuracy better than 40 meters. A detailed description of the data and study methodology is given in [1].

The `CNS` dataset contains the GPS trajectories of 689 individuals, recorded during the month of February 2014. In total, 1, 472, 296 data points are included, with a maximum temporal resolution of 15 minutes. The data density varies per individual and over time. On average, 2, 136 data points are recorded per user, corresponding to 76.3 data points per day. This means that for 79% of all 15-minute-segments, location data was recorded for the average user. If a time-segment is missing data, we use the value from the previous segment (thus assuming the user has not moved). We perform no additional processing to the data.

3.2 GEOLIFE dataset

The `GEOLIFE` dataset contains GPS trajectories collected in the Geolife project conducted by Microsoft Research Asia. The GPS signal was recorded using different GPS loggers and GPS-enabled phones. Participants were mostly students and faculty attending Beijing University. The study and its methodology is described in detail in [2, 3, 4].

The original dataset contains data of 178 users, collected over a period of four years (from April 2007 to October 2011). Due to the variety of data collection methods, the temporal resolution varies strongly in the original dataset: Most individuals produced the majority of their data in a small temporal window spanning multiple weeks or months, where their movements were recorded with a very high sampling rate of every 1 to 5 seconds. Conversely, there are long time spans where only few individuals generated data simultaneously.

To account for the uneven temporal sampling, we perform a supersampling of the data in order to increase the temporal density of the data, meaning that we map the periods with the highest data-density in the individual trajectories to the same global time span of one month. First, we resample the individual trajectories to a temporal resolution of 15 minutes, using the mean of latitude and longitude if multiple data points are registered in one segment. Then, for each individual, we determined their 37 day period with the most recorded datapoints. We keep only those individuals which have at least one data point on more than 90% of days in this 37 day period, leaving 75 of the original 178 users. For the remaining users, we determine the first Monday in this 37 day period, and finally use all data in the 30 days starting with this Monday at 0 am. as the individual data period $\mathcal{T}_i$. Finally, we gather all individual data periods $\mathcal{T}_i$ and map the timestamps to the same global 30 day period. This combined dataset is the dataset `GEOLIFE`. As all individual trajectories start on a Monday at midnight, the circadian and weekly rhythm of mobility is preserved.

The supersampling ensures that the dataset `GEOLIFE` has high-enough density to reliably find common locations among individuals and generate outbreak scenarios (the scenario generation is described in the main manuscript). However, we in our measurements we limit the maximum sample size to $N = 5$ individuals to avoid systematic biases. Increasing the sample size beyond $N = 5$ leaves very few locations

where this many individuals have gathered at any point in the dataset. We find that this limited set of locations, which are mostly living areas or lecture halls at Beijing university, introduces systematic biases in the data. For example, the error in determining the outbreak time Δt increases starting from $N = 4$, in contrast to all other datasets where the error decreases monotonously with sample size N (see Fig. 2 in main text). The increase in Δt is most likely due to the increasingly limited set of locations, which emphasizes locations that are visited regularly by a similar group of peoples, such as lecture halls.

The resulting GEOLIFE dataset contains 27,814 datapoints by 75 users. On average, there are 12.4 datapoints recorded per user per day, meaning that 12.9% of all 15-minute-segments contain data.

4 Synthetic datatsets

4.1 Exploration and preferential return model (dEPR dataset)

The dEPR dataset is created from simulations of the density-EPR (d-EPR) model. The d-EPR model is a variation of the EPR model (exploration and preferential return) which was first proposed in [5] and subsequently expanded to include spatial density in [6]. We use the default parameters as described in the original literature whenever possible. We will briefly recapitulate the model here and then describe the simulation algorithm used to generate the dataset.

Model description

In the d-EPR model, the mobility of an individual i follows two processes: One can either *return* to a previously visited location, or *explore* an new location. The probability for either process depends on the number of previously visited distinct locations $S(t)$ up to time t. The probability to explore a new location is

$$P_{\text{new}} = \rho S^{-\gamma}, \tag{1}$$

and the complementary probability to return to a previously visited location

$$P_{\text{ret}} = 1 - P_{\text{new}} = 1 - \rho S^{-\gamma}. \tag{2}$$

The parameter $0 < \rho < 1$ signifies the likelihood to explore at each step, and the parameter $\gamma \geq 0$ determines the rate at which new locations are explored: Say an

individual has performed n steps up to time t. As S increases by 1 each time a new location is explored, and thus $\mathrm{d}S/\mathrm{d}n = P_{\mathrm{new}}$, it follows that the number of visited location grows as $S \sim n^{1/(1+\gamma)}$.

When exploring a new location, the new location $j \neq i$ is chosen among a set of locations $\mathcal{G}$ with associated densities or frequencies g_j (which is the "density" extension of the original EPR model). Here, we use the locations obtained from Twitter data in the Berlin area (see section 5.1), where the frequency g_j is the number of Tweets counted at the location. Then, the probability to choose location j is given as

$$p_{ij} = \frac{1}{G}\frac{g_i g_j}{r_{ij}^2}, \tag{3}$$

where r_{ij} is the geographic distance between i and j and $G = \sum_{i,j\neq i} p_{ij}$ is a normalization constant.

When returning to a previously visited location, a location s is chosen randomly, proportional to the number of previous visitations f_s to each location. This *preferential return* mechanism results in a distribution of visitation frequencies that follows a Zipf's law,

$$f_k \sim k^{-\xi}, \tag{4}$$

where locations are sorted by rank k, which is often observed in empirical data [7].

Simulation algorithm

We generate the dataset `dEPR` by generating mobility trajectories $\{x_i(t)\}$ for a total of $N = 10,000$ individuals for a period of $T_{\max} = 30$ days in the following manner (the following description follows SI note 14 in [6]): Let $\mathcal{G}$ be the set of location obtained from Twitter data with associated frequencies g_i (see above). Starting at time $t = 0$, we place the individual at a location i chosen from the probabilities $p_i = \frac{1}{P} g_i$ with normalization $P = \sum_i g_i$. The number of locations visited is $S = 1$. Then, we follow these steps:

1 We draw the waiting time Δt, that is the time until the next mobility event takes place, from a power-law distribution with an exponential cutoff, $P(\Delta t) \sim \Delta t^{-1-\beta} e^{(\Delta t/\tau)}$. We use the parameters $\beta = 0.8$ and $\tau = 17$ hours as measured in the original EPR-study [5]. The random numbers for the waiting time are

generated using the process described in algorithm 5 in [7]. We update the time to $t \rightarrow t + \Delta t$.

2 We calculate the probability $P_{\mathrm{new}} = \rho S^{-\gamma}$ that an individual explores a new location, where we use $\rho = 0.6$ and $\gamma = 0.21$ as in [5]. If exploration is chosen, continue at step 3, otherwise at step 4.

3 If the individual at location i explores, the new location j is selected according the probabilitys p_{ij} following the gravity law eq. 3. We increase the number of distinct locations visited, S, by one.

4 If the individual returns to a previous location, we choose one location j among the set of previously visited locations, proportional to the number of previous visits f_j.

5 We add the spatial location x_j at time t to the movement trajectory $\{x_i(t)\}$ and return to step 1, unless $t \geq T_{\mathrm{max}}$.

Dataset

The resulting `dEPR` dataset contains $2,248,177$ datapoints contributed by $10,000$ individuals over a timespan of 30 days. On average, there are 224 datapoints per individual, or 7.49 datapoints per individual per day, meaning that an individual changes its location.

4.2 OD simulations (sOD and dOD datasets)

Model description

The datasets `sOD` and `dOD` are generated with agent based simulation based on origin-destination (OD) mobility data. The mobility data we use is extracted from mobile phone cell tower logs in the area of Berlin, Germany. The area is divided into a total of L spatial cell $i = 1, ..., L$. We obtain the *OD matrix* $\mathcal{F}(t)$, where each entry $F_{ij}(t)$ is the number of all recorded trips originating in spatial cell i and ending in spatial cell j within a certain time period (see section 5.2 for a more detailed description of how the mobility data is collected and how $\mathcal{F}(t)$ is constructed). From the absolute number of trips in the OD matrix $\mathcal{F}(t)$ we construct the *transition probability matrix* $\mathcal{P}$, where

$$P_{ij}(t) = \frac{F_{ij}(t)}{\sum_j F_{ij}(t)}$$

is the probability that a trip that started in spatial cell i at time t ended in cell j.

In our model, we assume that individuals transition between spatial cells according to the probabilities P_{ij}. The datasets `sOD` and `dOD` differ in how the exact latitude-longitude location within the spatial cells is chosen: For the dataset `sOD` (spatial-OD), the location is chosen randomly within the bounds of the spatial cell. For the dataset `dOD` (density-OD), the location is chosen from the locations extracted from Twitter that fall within the spatial cell, proportional to the frequency of Tweets at the location.

Simulation algorithm

The datasets `sOD` and `dOD` each consist of mobility trajectories of $N = 10,000$ individuals over a period of $T_{\max} = 30$ days that are generated in the following way:

Starting at time $t = 0$, we place the individual in a starting cell i, which is chosen randomly proportional to the total flux F_i that originates from each cell over all observation times t_k,

$$F_i = \sum_k \sum_j F_{ij}(t_k)$$

that is the starting cell i is chosen according the probabilities

$$p_i = F_i / \sum_j F_j$$

. Within the starting cell i, the individual is placed

- for `sOD`: At a random latitude-longitude coordinate x_m within the spatial bounds of cell i.
- for `dOD`: At the coordinates x_m one of the locations $\mathcal{G}_i$, which is the subset of all the locations $\mathcal{G}$ extracted from Twitter data that fall into the spatial bounds of cell i (see sec. 5.1 for details on the Twitter dataset). The location is chosen randomly from $\mathcal{G}_i$, proportional to the number of Tweets g_i at the location. If none of the locations in $\mathcal{G}$ are within the spatial bounds of cell i, the coordinates are chosen randomly within cell i.

Then, we follow the following steps:

1 We draw the waiting time Δt, that is the time until the next mobility event takes place, from a power-law distribution with an exponential cutoff, same as detailed for the `dEPR` model described above. We update the time to $t \to t+\Delta t$.

2 We draw the target spatial cell j from the transition probabilities $P_{ij}(t)$.

3 Within the spatial cell j, we choose the exact spatial coordinates x_m as detailed for the starting cell above.

4 We add the location x_m at the time t to the movement trajectory $\{x_i(t)\}$ and return to step 1, unless $t \geq T_{\max}$.

Dataset

The statistics of the `sOD` `dOD` datasets are very similar to the `dEPR` dataset, as large parts of the methodology are the same. Both datasets contain roughly 2.25 million datapoints divided among $10,000$ individuals, corresponding to 7.49 datapoints per individual per day.

5 Auxilliary datasets

5.1 Berlin twitter dataset

The dataset `tweets-berlin` comprises locations that were extracted from geo-located posts on Twitter in the area of Berlin. The dataset is used in the dEPR and dOD models, where one requires a spatial density distribution of locations, that is a set of locations G with associated visitation frequencies g_i. We obtain these locations G from geo-located posts from Twitter, which we will describe in the following. The resulting dataset `tweets-berlin` is made available in the OSF repository.

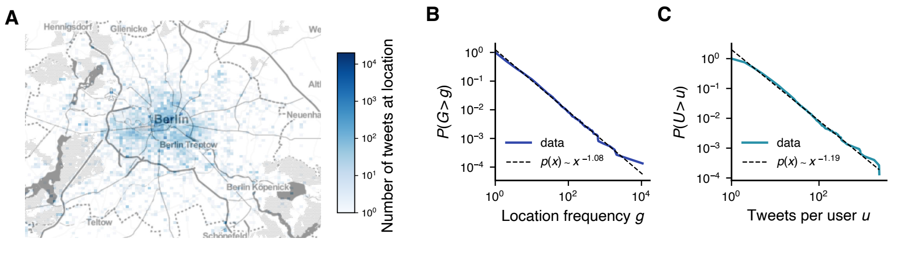

Figure 3 Spatial distribution and statistics of the Twitter dataset. A) Spatial distribution of the $63,607$ posts in the dataset. B) The number of posts at each location and the B) number of posts per user follow power laws, a characteristic feature of geo-located mobility data [7].

We collected posts published on Twitter during January to December 2018, using the Twitter API v1 [8] and the python package `tweepy` [1]. Among all posts, we only collected those posts that contained a geo-tag, that is a geo-location as measured by the device (mostly via GPS). Around 0.85% of all posts include such a geo-tag [9]. Furthermore, we only collect posts in the area of Berlin, which we define as geo-tagged posts within the bounds of longitude $13.1 - 13.7$ and latitude $52.3 - 52.7$, encompassing an area of roughly 60 km width and height. For each post, we save the time, user id, and longitude and latitude coordinates.

We perform some additional manual processing to improve the data quality. Many of the users with the most posts are not human, but automated systems or bots — for example webcams, weather stations, or radio stations announcing the current song. We removed such users from the data by going through the 100 users with the most posts, manually identifying non-human users based on the content of their posts, and removing all such users from the dataset.

From the collected posts, we extract a set of locations $\mathcal{G}$ by clustering the spatial coordinates of the posts. Clustering is a common method used to identify unique locations from geolocated data [10]. Posts that come from the same locations are likely to have small variations in the longitude-latitude coordinates, despite representing the same location. By clustering posts in close spatial vicinity, one can identify unique locations and correctly count the number of posts there. We use the density-based `DBSCAN` algorithm for the spatial clustering, which is a common choice for this task [11]. The algorithm has one main parameter, which is the maximum distance between two posts at which they can still be considered part of the same location, which we set as 100m, which is reasonable in our context and the default choice in the `scikit-mobility` Python package [10]. Finally, we define the spatial coordinates of each location identified by the clustering as the average longitude and latitude coordinates of all posts associated with the location.

The resulting dataset `tweets-berlin` contains $63,607$ posts by $7,493$ unique users. Among these posts, we identify $7,436$ unique locations by clustering, which we use as the set of locations $\mathcal{G}$ in the mobility models. The location frequency g_i is the number of associated posts at that location. Figure 3 shows the spatial distribution and some characteristic statistics of the dataset.

---

[1] https://www.tweepy.org/

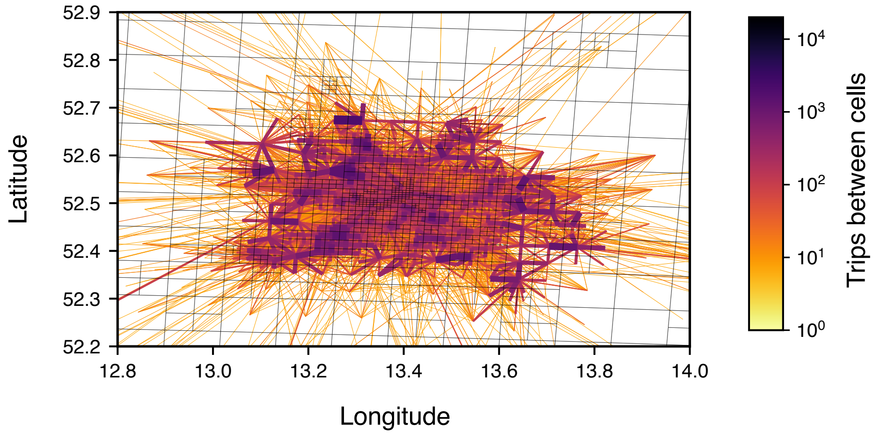

Figure 4 Illustration of the Origin-Destination matrix. The spatial area of Berlin is divided into cells of varying size. Here, we show the total number of trips between cells in an "average week" of Q4 2018 (see text for details). The color and width of lines indicate the number of trips between cells.

5.2 OD dataset

In the mobility models we employ and origin-destination matrix $\mathcal{F}$, which we obtained from call-data records of a mobile phone provider for the area of Berlin (see an illustration in Fig. 4). The dataset `ODdata` is collected from the 46 million German customers of the provider. Included are all trips that either started or ended within the district of Berlin. A trip is recorded when a device leaves its current cell tower area A, passes through one or multiple other towers, until it becomes stationary again in cell tower area B. This is counted as one trip from cell A to B. It is assumed that a device is "stationary" if no movement is recorded for approximately 15 minutes. The start- and end-areas A and B can be the same, meaning that self-loops are recorded as well.

The trips recorded on the level of cell towers are subsequently aggregated into spatial cells i, which range in size from 500m $\times$ 500m in the city center to 5km $\times$ 5km in more rural areas, depending on cell tower density (see Fig. 4). Our dataset encompasses 780 spatial cells, which are either cells within the district of Berlin, or cells where trips from or to such Berlin-cells started or ended, respectively.

Trips are also temporally aggregated, in hourly bins. Specifically, the data we use was collected during the Q4 of 2018, and averaged to one "average week": For

each hour and each day from Monday to Sunday (for example, Monday 8-9am), the number of trips within this time slot was averaged over all instances of this time slot (all Mondays 8-9 am within Q4 2018). Note that a trips is attributed to a time slot when the start time of the trip falls within the slot, and the end time is disregarded.

As a result, we obtain the origin-destination matrix $\mathcal{F}$, where each entry $F_{ij}(t)$ denotes the number of trips from spatial cells i to j at time t, where t ranges in hourly increments from Monday 0 am to Sunday 12 pm. In the simulations of the mobility models, we employ periodic boundary conditions to simulate trajectories longer than one week, i.e. $\mathcal{F}(t + 7\text{days}) := \mathcal{F}(t)$. The matrix $\mathcal{F}$ includes a total of $6,945,418$ trips, or an average number of $41,341$ trips starting in each hourly bin. The average number of trips between (or within) the 780 spatial cells in each hourly bin is 234.

6 Data availability

The synthetic datasets `dEPR`, `sOD` and `dOD` are available in the OpenScienceFramework (OSF) repository https://osf.io/3rzh8/. These each include the simulated mobility trajectories of $10,000$ individuals over 30 days at a resolution of 15 minutes. The two auxilliary datasets `tweets-berlin` and `ODdata`, which are used for the mobility simulations, are also made available in the OSF repository.

The `GEOLIFE` dataset collected by Microsoft Research is available online and linked in the original study [2, 3, 4]).

The `CNS` dataset was provided to the authors for the purpose of this study by the original authors of the Copenhagen Network Study; please refer to them for requests for access to the data [1].

Author details

[1]Robert Koch Institute, Nordufer 20, 10115, Berlin, Germany. [2]Humboldt University Berlin, Dummy, 24105, Berlin, Germany.

References

1. Stopczynski, A., Sekara, V., Sapiezynski, P., Cuttone, A., Madsen, M.M., Larsen, J.E., Lehmann, S.: Measuring large-scale social networks with high resolution. PloS one **9**(4), 95978 (2014). doi:10.1371/journal.pone.0095978
2. Zheng, Y., Li, Q., Chen, Y., Xie, X., Ma, W.Y.: Understanding mobility based on GPS data. In: Proceedings of the 10th International Conference on Ubiquitous Computing. UbiComp 2008, pp. 312–321, Seoul, South Korea (2008). doi:10.1145/1409635.1409677

3. Zheng, Y., Zhang, L., Xie, X., Ma, W.Y.: Mining interesting locations and travel sequences from GPS trajectories. In: Proceedings of the 18th International World Wide Web Conference. WWW'09, pp. 791–800, Madrid, Spain (2009). doi:10.1145/1526709.1526816
4. Zheng, Y., Xie, X., Ma, W.: GeoLife: A Collaborative Social Networking Service among User, Location and Trajectory. IEEE Data Eng. Bulletin **33**(2), 32–40 (2010). doi:10.1.1.165.4216
5. Song, C., Koren, T., Wang, P., Barabási, A.L.: Modelling the scaling properties of human mobility. Nature Physics **6**(10), 818–823 (2010). doi:10.1038/nphys1760
6. Pappalardo, L., Simini, F., Rinzivillo, S., Pedreschi, D., Giannotti, F., Barabási, A.L.: Returners and explorers dichotomy in human mobility. Nature Communications **6**, 1–8 (2015). doi:10.1038/ncomms9166
7. Barbosa, H., Barthelemy, M., Ghoshal, G., James, C.R., Lenormand, M., Louail, T., Menezes, R., Ramasco, J.J., Simini, F., Tomasini, M.: Human mobility: Models and applications. Physics Reports **734**, 1–74 (2018). doi:10.1016/j.physrep.2018.01.001. 1710.00004
8. Twitter: Twitter API Documentation
9. Sloan, L., Morgan, J.: Who tweets with their location? Understanding the relationship between demographic characteristics and the use of geoservices and geotagging on twitter. PLoS ONE **10**(11), 0142209 (2015). doi:10.1371/journal.pone.0142209
10. Pappalardo, L., Simini, F., Barlacchi, G., Pellungrini, R.: scikit-mobility: a Python library for the analysis, generation and risk assessment of mobility data (2019). 1907.07062
11. Ester, M., Kriegel, H.-P., Sander, J., Xu, X.: A Density-Based Algorithm for Discovering Clusters in Large Spatial Databases with Noise. In: Kdd vol. 96, (1996). www.aaai.org
